# Datasets supporting the adoption of multifunctional cover crops related to soil water and nitrogen in water-limited environments

**DOI:** 10.1016/j.dib.2022.108841

**Published:** 2022-12-19

**Authors:** Ismail Ibrahim Garba, Alwyn Williams

**Affiliations:** a School of Agriculture and Food Sciences, The University of Queensland, Gatton, QLD 4343, Australia; b Centre for Dryland Agriculture, Bayero University Kano, PMB 3011 Kano, 70001, Nigeria

**Keywords:** Conventional fallow, Crop rotation, Ecosystem services, Dryland, Multifunctionality

## Abstract

Crop diversification with cover crops could deliver a wide range of agroecosystem services including water conservation, nutrient cycling, biodiversity, and crop productivity as well as reducing the negative environmental footprint of conventional fallows. However, the potential competition of cover crops and subsequent cash crops on plant available water and soil mineral nitrogen (N) has limited the adoption of cover cropping for fallow replacement in water-limited environments. This article provides datasets for understanding the multifunctional role of cover crops as an alternative paradigm to conventional fallow in water-limited environments. The dataset is divided into four components comprising measured cover crop parameters (21 variables, *n* = 144), soil water (4 variables, *n* = 2,159), soil mineral N (4 variables, *n* = 1440), and site characteristics (8 variables). The datasets consist of crop resource quantity (biomass, N uptake, δ¹⁵N (‰), fixed shoot N, and water use), resource quality (C/N ratio, lignin, acid detergent fibre, and N concentration), and soil status (plant available water and mineral N) at field scale. The data supports the framework of the continuous development of alternative innovative cropping systems that have the potential to increase and maintain crop yield while minimizing the adverse effects of conventional fallowing in the context of sustainable intensification. The datasets are associated with the original research article published in *Agriculture, Ecosystem, and Environment* entitled “Fallow replacement cover crops impact soil water and nitrogen dynamics in a semi-arid sub-tropical environment” as Garba et al. [1].

**Specifications Table**

**Table:** 

Subject	Agronomy and Crop Science.
Specific subject area	Conventional fallow; crop rotation; ecosystem services; dryland; multifunctionality.
Type of data	Table Figure Excel
How the data were acquired	In-situ field measurements of crop and soil parameters. In addition, plant and soil samples were collected from a field experiment and analyzed for the various parameter in a laboratory.
Data format	Raw Analyzed
Description of data collection	Soil samples were collected at different times in a typical crop-fallow rotation and years at various depths (0-10cm, 10-30cm, 30-60cm, 60-90cm, and 90-120cm). The samples were processed at a laboratory using standard protocols and further analyzed by wet chemistry. Plant samples were collected from 4 cover crop species of varying functional trait diversity utilizing Poaceae [forage oat], Fabaceae [common vetch or fababean] and Brassicaceae [forage rape] families), a conventional fallow and stubble treatment. The samples were analyzed for quality parameters following standard processing.
Data source location	• The University of Queensland: • UQ Crop Research Unit, Gatton, Queensland: • Australia: Latitude: 27°32′09.6"S Longitude: 152°20′26.6"E, Altitude: 89 m
Data accessibility	Repository name: The University of Queensland eSpace Data identification number: 8c41f39 Direct URL to data: 10.48610/8c41f39
Related research article	I.I. Garba, D. Fay, R. Apriani, Pg. Dk Yusof, D. Chu, A. Williams, Fallow replacement cover crops impact soil water and nitrogen dynamics in a semi-arid sub-tropical environment, Agric. Ecosyst. Environ. 338 (2022) 108052. 10.1016/j.agee.2022.108052.

## Value of the Data

• This dataset provides baseline data for quantitative assessment of the impact of fallow replacement cover crops on soil water and mineral N in a globally important dryland grain production region.
• The trade-offs between cover crop ecosystem services (improving fallow efficiency and N cycling) and disservices (soil water and N use) have limited cover crop adoption in drylands. This dataset provides insights for finding the balance between ecosystem services and tradeoffs in water and N use toward addressing some of the barriers to cover crop adoption in dryland.
• This dataset may be useful to develop cover crop best management practices for dryland region that is currently lacking. For example, by providing benchmarks of cover crop water use and groundcover dynamic towards stabilizing crop yields and minimizing negative environmental footprints of conventional fallows.
• The methodology is innovative and can be used as a guide for developing a framework or evaluating cover crop multifunctionality based on mixture experiments.

## 1 Data Description

**Fig. 1:**
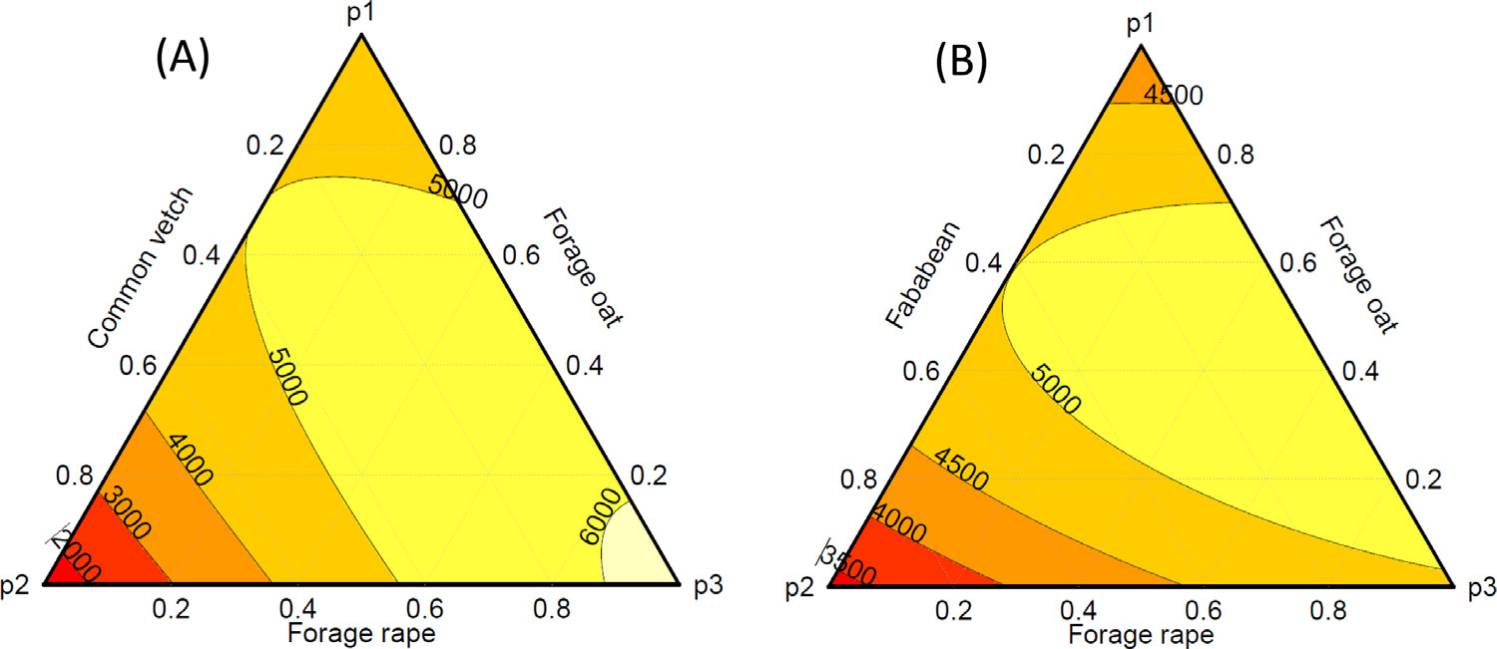
Contour plot of cover crop biomass production in 2020/2021 (A) and 2021/2022 (B) comprising the three cover crop functional types: Poaceae [forage oat (*Avena sativa* L., cv. Comet)]; Brassicaceae [forage radish (*Brassica napus* L., cv Greenland SF)]; and Fabaceae [common vetch (*Vicia sativa* subsp. *sativa* L., cv. Morava) or fababean (*Vicia faba* L., cv. Nasma)]. The p1, p2, and p3 are the sowing proportion of each cover crop species and are indicated in the raw datasets1-3. The proportions showed on the graph were the sowing treatment identity (Oat: Common vetch/Fababean: Rape) as 1 = Fallow; 2 = Stubble; 3 = 100:0:0; 4 = 0:100:0; 5= 0:0:100; 6 = 50:50:0; 7= 50:0:50; 8 = = 0:50:50; 9 = 33:33:33; 10 = 70:15:15; = 15:70:15; 12 = 15:15:70.

**Fig. 2:**
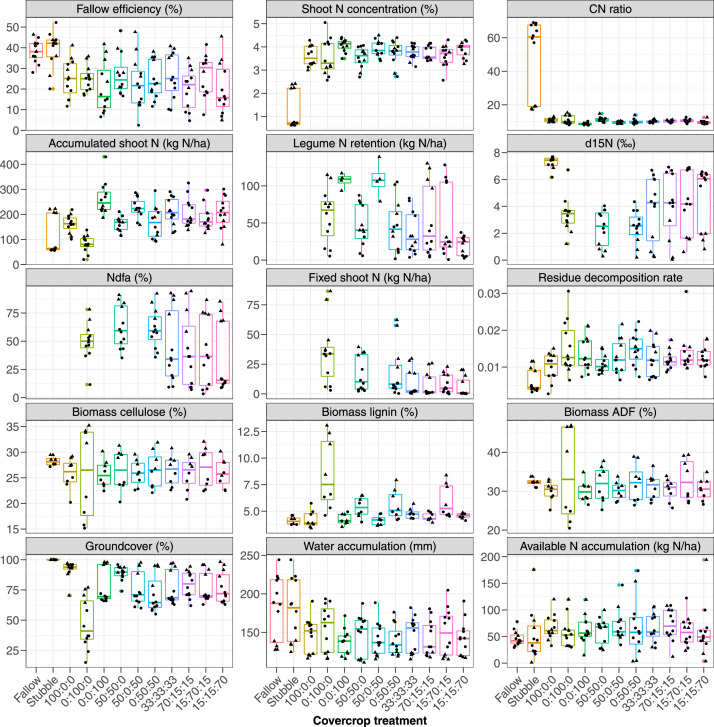
Fallow efficiency (%), cover crop shoot N concentration (%), residue C/N ratio, total accumulated biomass N (kg N ha^−1^), the fraction of legume shoot N kg N ha^−1^), the proportion of N derived from biological N fixation (Ndfa, %), fixed shoot N kg N ha^−1^), residue decomposition constant, cellulose content (%), lignin content (%), acid detergent fibre content (ADF, %), cover crop groundcover at termination (%), crop water use (mm), and aggregated profile (0-120 cm) available N accumulated from cover crop termination to cash crop sowing (kg N ha^−1^). The circle showed 2020/2021 and the triangle showed the 2021/2022 data of the different cover crops and the control treatments. The proportions showed on the graph were the sowing treatment identity (Oat: Common vetch/Fababean: Rape) as 1 = Fallow; 2 = Stubble; 3 = 100:0:0; 4 = 0:100:0; 5= 0:0:100; 6 = 50:50:0; 7= 50:0:50; 8 = = 0:50:50; 9 = 33:33:33; 10 = 70:15:15; = 15:70:15; 12 = 15:15:70.

**Fig. 3:**
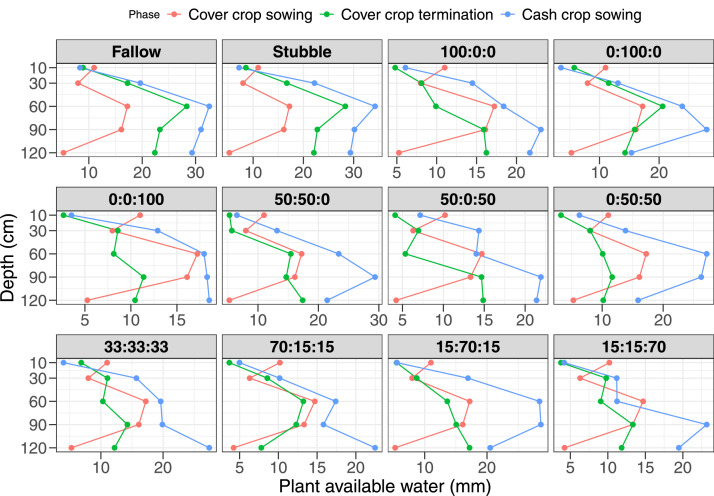
Plant available water content (mm) estimated as the difference between crop drained upper limit at 0.1bars (DUL) and crop lower limit at 15 bars at cover crop sowing, termination, and cash crop sowing in 2020/2021 of the different cover crop treatments (forage oat: common vetch: forage rape). The proportions showed on the graph were the sowing treatment identity (Oat: Common vetch/Fababean: Rape) as 1 = Fallow; 2 = Stubble; 3 = 100:0:0; 4 = 0:100:0; 5= 0:0:100; 6 = 50:50:0; 7= 50:0:50; 8 = = 0:50:50; 9 = 33:33:33; 10 = 70:15:15; = 15:70:15; 12 = 15:15:70.

**Fig. 4:**
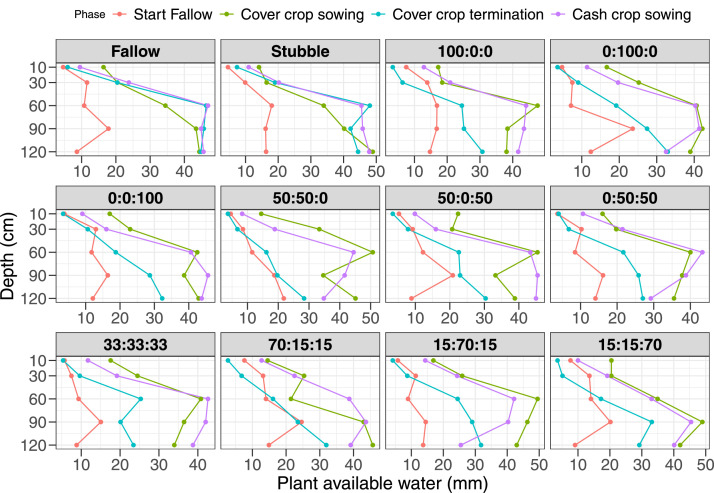
Plant available water content (mm) estimated as the difference between crop drained upper limit at 0.1bars (DUL) and crop lower limit at 15 bars at beginning of fallow (cash crop harvest), cover crop sowing, termination, and cash crop sowing in 2021/2022 of the different cover crop treatments (forage oat: fababean: forage rape). The proportions showed on the graph were the sowing treatment identity (Oat: Common vetch/Fababean: Rape) as 1 = Fallow; 2 = Stubble; 3 = 100:0:0; 4 = 0:100:0; 5= 0:0:100; 6 = 50:50:0; 7= 50:0:50; 8 = = 0:50:50; 9 = 33:33:33; 10 = 70:15:15; = 15:70:15; 12 = 15:15:70.

**Fig. 5:**
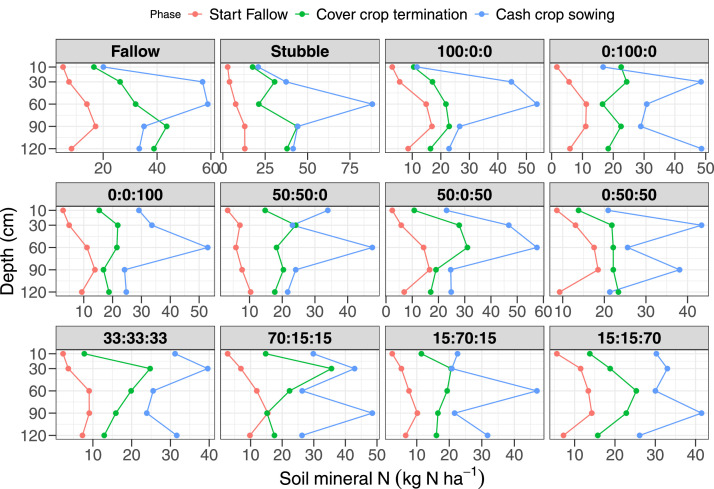
Soil mineral N (kg N ha^−1^) at the beginning of fallow, cover crop termination, and cash crop sowing in 2020/2021 of the different cover crop treatments (forage oat: common vetch: forage rape). The proportions showed on the graph were the sowing treatment identity (Oat: Common vetch/Fababean: Rape) as 1 = Fallow; 2 = Stubble; 3 = 100:0:0; 4 = 0:100:0; 5= 0:0:100; 6 = 50:50:0; 7= 50:0:50; 8 = = 0:50:50; 9 = 33:33:33; 10 = 70:15:15; = 15:70:15; 12 = 15:15:70.

**Fig. 6:**
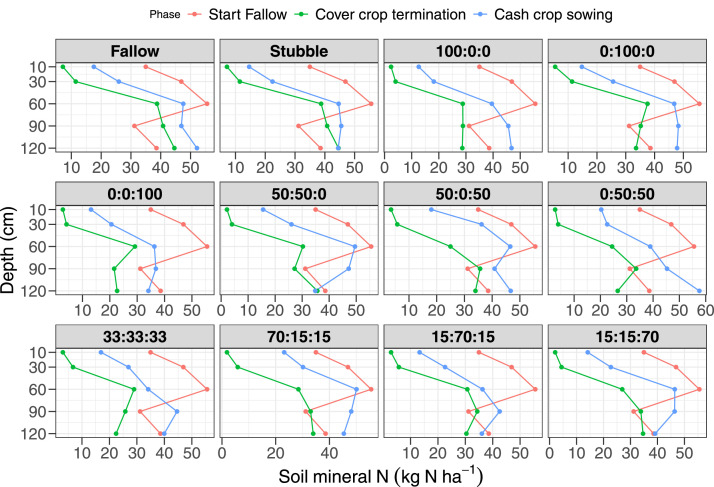
Soil mineral N (kg N ha^−1^) at the beginning of fallow, cover crop termination, and cash crop sowing in 2021/2022 of the different cover crop treatments (forage oat: common vetch: forage rape). The proportions showed on the graph were the sowing treatment identity (Oat: Common vetch/Fababean: Rape) as 1 = Fallow; 2 = Stubble; 3 = 100:0:0; 4 = 0:100:0; 5= 0:0:100; 6 = 50:50:0; 7= 50:0:50; 8 = = 0:50:50; 9 = 33:33:33; 10 = 70:15:15; = 15:70:15; 12 = 15:15:70.

This dataset comprises descriptive (mean and standard error) plant and soil data on the impact of cover crop functional trait diversity and sowing proportion adjustment of cover crop mixtures on soil functions, and crop productivity. This ongoing research was designed to provide information on where cover crops fit “best” into dryland crop-fallow rotation by manipulation of cover crop functional diversity (grass vs. legumes vs. brassica) on soil functions (soil water retention, aggregates stability, soil C and N cycling), soil health (soil respiration, microbial community abundance and composition and suppression of parasitic nematodes). The dataset is divided into four components comprising cover crop (21 variables, *n* = 144), soil water (4 variables, *n* = 2,159), soil mineral N (4 variables, *n* = 1440), and site characteristics (8 variables). The variables and their units are provided in Table 1. The raw datasets (Dataset1-3) used to produce Fig. 1-6 and additional description of the measured variables are available in Supplementary material S1 and publicly available at The University of Queensland eSpace data repository (10.48610/8c41f39). The first dataset (Dataset1_Covercrop) comprised the cover crop component of the data and includes cover crop biomass production in kg DM ha^−1^ (Fig. 1), cover crop N uptake in kg N ha^−1^ (ASN) and proportion (%DFA) and amount of N fixed via biological N fixation (BNF) in kg N ha^−1^, cover crop residue quality (CN ratio, lignin (%), acid detergent fibre (%ADF), cellulose (%), and N concentration (%) in biomass) as well as total soil water accumulation during fallow (mm) as shown in Fig. 2. The Fig. 1 represented the biomass production of each cover crop functional type (OatDMkg_ha, VetchDMkg_ha, and RapeDMkg_ha) in monoculture and mixture cropping as a contour plot based on the sowing proportions of each cover crop species in each treatment. The soil water data consisted of plant available water (PAW) at 0-10, 10-30, 30-60, 60-90, and 90-120 cm soil depth measured at the start of fallow, cover crop sowing, cover crop termination and cash crop sowing (Raw dataset Dataset2_Soil_water) for Mendel and T-block in 2020/2021 (Fig. 3) and 2021/2022 (Fig. 4). The Dataset D3_Soil_nitrogen showed the measured soil mineral N (NH_4_-N + NO_3_-N) adjusted for layer thickness (cm) and bulk density at 0-10, 10-30, 30-60, 60-90 and 90-120 cm soil depth measured at the start of fallow, cover crop termination and cash crop sowing for Mendel and T-block in 2020/2021 (Fig. 5) and Mendel site only in 2021/2022 (Fig. 6).

**Table 1:** Key variables measured and their units

Category	Variable	Description	Unit	Method of determination
Cover crop	OatDMkg_ha	Forage oat biomass dry matter	kg DM ha^−1^	Destructive plant cut
Cover crop	VetchDMkg_ha	Legume (common vetch or fababean) biomass dry matter	kg DM ha^−1^	Destructive plant cut
Cover crop	RapeDMkg_ha	Forage rape biomass dry matter	kg DM ha^−1^	Destructive plant cut
Cover crop	CCDMkg_ha	Total cover crop biomass dry matter	kg DM ha^−1^	Destructive plant cut
Cover crop	WeedDMkg_ha	Weed biomass dry matter	kg DM ha^−1^	Destructive plant cut
Cover crop	TotalDMkg_ha	Total aboveground biomass dry matter	kg DM ha^−1^	Destructive plant cut
Cover crop	GCR	Cover crop groundcover at termination from UAV flights	%	Estimated as Optimized Soil Adjusted Vegetation Index (OSAVI) from UAV flights
Cover crop	CEP	Cover crop water use	mm	Estimated
Cover crop	Percent C	Biomass C content	%	Dumass combustion in LECO C/N analyzer
Cover crop	NCR	Biomass N content	%	Dumas combustion in LECO C/N analyzer
Cover crop	CNR	Biomass C/N ratio	-	Estimated from Percent C and NCR
Cover crop	ASN	Accumulated shoot N	kg N ha^−1^	Estimated using NCR and CCDMkg_ha
Cover crop	LNF	Fraction of legume biomass in mixtures	kg N ha^−1^	Estimated
Cover crop	NAT	δ¹⁵N (‰) determined via the natural abundance method	-	natural abundance method
Cover crop	DFA	The proportion of BNF derived N	%	Estimated from NAT
Cover crop	BNF	Fixed shoot N	kg N ha^−1^	Estimated from DFA and VetchDMkg_ha
Cover crop	DER	Residue decomposition constant (λ)	-	Estimated from negative exponential decay model
Cover crop	CEL	Cellulose content	%	Gravimetric technique in an automated fiber analyzer
Cover crop	LIG	lignin content	%	Gravimetric technique in an automated fiber analyzer
Cover crop	ADF	acid detergent fibre	%	Gravimetric technique in an automated fiber analyzer
Cover crop	FEE	Fallow efficiency	%	Estimated
Cover crop	MIN	Mineral N accumulation from cover crop termination to cash crop sowing	kg N ha^−1^	Estimated
Soil water	Thickness (cm)	Layer thickness	cm	Measured
Soil water	vwc	Volumetric water content	mm^3^ mm^−3^	Estimated
Soil water	ll5	Crop lower limit determined using pressure plates at 15bars	mm	Pressure plate
Soil water	paw	Plant available water (vwc-ll5) adjusted for layer thickness and BD	mm	Estimated
Soil nitrogen	NH4-N	NH4-N	mg kg^−1^	Colourimetric assay following with 2 M KCl extraction
Soil nitrogen	NO3-N	NO3-N	mg kg^−1^	Colourimetric assay following with 2 M KCl extraction
Soil nitrogen	SMN (kgN_ha)	Soil mineral N (NH_4_-N+NO_3_-N) adjusted for layer thickness and BD	kg N ha^−1^	Estimated

## 2 Experimental Design, Materials and Methods

### 2.1 Experimental design and field procedures

The ongoing research commenced in 2020 at The University of Queensland Crop Research Station across two sites with contrasting management histories (Mendel block and T-block). The region has a semi-arid subtropical climate with a long-term (1989-2019) average annual precipitation of 697 mm predominantly received in the summer. The Mendel site had a black deep-cracking self-mulching vertosol soil with plant available water capacity (PAWC) of 215 mm for 0-120 cm soil profile depth and was under summer fallow before the trial establishment. The T-block soil was a dark grey vertosol with PAWC of 202 mm in the 0-120 cm soil profile depth and was under long fallow and summer mungbean before the establishment of the current research. More detailed information is available from the original research article published as Garba et al. [1].

The trial consisted of 12 treatments comprising 10 cover crop sowing proportions in simplex centroid design, a conventional fallow (control), and a high C/N ratio stubble (local check) with four replications in each site. The plot factor was cover crop consisting of three taxonomic groups: Poaceae [forage oat (*Avena sativa* L., cv. Comet)]; Brassicaceae [forage rape (*Brassica napus* L., cv Greenland SF)]; and Fabaceae [common vetch (*Vicia sativa* subsp. *sativa* L., cv. Morava)]. The common vetch was replaced with fababean (*Vicia faba* L., cv. Nasma) in the 2021/2022 season due to poor growth in 2020/2021. The sowing proportions were: three 100% monocultures of each cover crop species, three 50:50% proportion combinations, one even three-species mixture (33:33:33), and three replacement series of each species (70:15:15 combinations) as shown in Fig. 1. The sowing proportions were applied as an adjustment to the standard (100%) seeding rate. The standard seeding rates were 40 kg seeds ha^−1^ for forage oat, 40 kg seeds ha^−1^ common vetch, 200 kg seeds ha^−1^ fababean and 4 kg seeds ha^−1^ for forage rape, respectively. The conventional fallow treatment was included as a control where no plants were grown over the entire winter period while the high C/N ratio (64:1 in 2020/2021 and 18:1 in 2021/2022) residue stubble (fully matured oat/barley hay at 10t DM ha^–1^) was included to serve as local checks to estimate the extreme ends of groundcover and N immobilization effect on soil water and N dynamics. The stubble treatment was maintained identically to the conventional fallow as chemical fallow and the residue was only applied after cover crop termination. The experiments were laid out in 8 m × 5 m plots and 2 m alleyways between plots and replicates in each site. Before cover crop sowing, the sites were incorporated with starter 30kg ha^−1^ GranulockZ fertilizers containing 11 % N, 21.8 % P, 4 % S, and 1% Zn (Incitec Pivot Fertilisers, Melbourne) to ensure there were no soil nutrient limitations at the start of the experiment. The cover crops were seeded into 12 rows using a double-disc planter on a 35 cm row spacing. There was a drought in 2020/2021, therefore, supplementary irrigation was provided to ensure cover crop growth and it was applied to bring the water input to within 85% long-term average precipitation of the experimental sites.

### 2.2 Plant measurements

Weekly estimates of groundcover were monitored using the DJI Phantom 4 Pro unmanned aerial vehicle (UAV system) equipped with a red-green-blue (RGB) camera with a 20-megapixel resolution. Each flight was executed with 75% side and 80% front overlap in clear sky conditions at 40 m altitude. The groundcover was determined as the Optimized Soil Adjusted Vegetation Index (OSAVI) vegetation index following the processing of the raw images and then stitched to obtain an accurate orthomosaic and then segmented according to plot layout using the PhenoCopter system [2].

The cover crops were terminated at forage oat Zadok 32 (late stem elongation) about 90 days after sowing in 2020/2021 and 80 days after sowing in 2021/2022. The termination was with using glyphosate (450 g L^−1^ isopropylamine) at 2.4 L ha^−1^ and Sharpen (700 g kg^−1^ saflufenacil) plus 1% v/v Hasten adjuvant.

Before the desiccation, cover crop biomass was sampled at ground level from the 1 m^2^ quadrat (middle four rows) of each plot, partitioned into component crops and weeds, and oven-dried at 65 ^°^C until constant weights to estimate total dry matter production. The dried biomass samples were then bulked for each and ground to 2 mm for subsequent total biomass N (%), C (%), acid detergent fiber (ADF, %), lignin (%), and cellulose (%) content analyses at the Chemical Analytical Laboratory of The University of Queensland. The biomass C (%) and N (%) were determined by Dumas combustion and analysis in a LECO C-N analyzer (CN 928 Series, LECO Corporation, The Netherlands) as described in Rayment et al [3]. The ADF (%), lignin (%), and cellulose (%) content were estimated by gravimetric technique in an automated fiber analyzer (Fibertech TM 8000, FOSS, Denmark), using the modified methods of Soest [4]. Accumulated shoot N (kg N ha^−1^) was determined as the product N concentration (%) in biomass and cover crop aboveground biomass (kg ha^−1^).

To determine the residue decomposition dynamics of the cover crop residues, a modified litter bag technique [5,6] was utilized. Special litter bags were prepared using Cyclone PVC coated fiberglass screen with a mesh size of 2.5 mm to sew to form seven pieces of 0.57 m × 0.25 m sufficient to cover 1 m^2^. The cover residue from the 1 m^2^ quadrat and the stubble were evenly divided into seven aliquots, and each was placed into separate litter bags and returned to the plots. A Litter bag was retrieved from each plot at 20, 40, 60, 90, 120, and 150 days after termination (DAT) to determine the percentage of mass or N remaining (DM, % N content) at each retrieval time. At each sampling, the litter bags were screened for foreign materials (soils, etc) and oven-dried at 65°C and then weighed for contamination by ashing a subsample in a muffle furnace at 550°C for 6 hours and adjusting the residue DM, total C, and N contents to an ash-free basis. The percentage residue mass remaining (%DM) was fitted to a negative exponential equation [7] as shown in Eq. (1):

(1) $$M_{t}=M_{o}e^{-\lambda t}$$

Where; *M_t_* = cover crop residue DM remaining at time *t, M_0_* = cover crop residue DM remaining at zero days after cover crop termination, *λ* = decay constant, *t* = days after cover crop termination, and *e* = base of natural logarithms. The residue decomposition constant (DER) was reported as the decay constant (*λ).* The exponential decomposition models have been extensively used to describe residue decomposition in litter bags [6,^9^,10].

To determine the N contribution of the legume cover crops via biological N fixation, the δ^15^N natural abundance method [8] was utilized by grinding a sub-sample of the common vetch/fababean and the forage oat biomass to 0.5 mm and analyzed at the Stable Isotope Facility, University of California, Davis using PDZ Europa ANCA-GSL elemental analyzer interfaced to a PDZ Europa 20–20 continuous isotope mass spectrometer (IRMS) (Sercon Ltd., Cheshire, UK). The proportion of the N-derived from BNF (%Ndfa) was estimated using Eq. 2.

(2) $$\%\;Ndfa=\;\frac{\delta^{15}N_{ref}-\delta^{15}N_{legume}}{\delta^{15}N_{ref}-\beta }\;\times \;100$$

where *δ^15^N_ref_* is the *δ^15^N* signature of the reference plant, *δ^15^N_legume_* is the *δ^15^N* signature of the legume (common vetch or fababean), and β is defined as the *δ^15^N* signature of the legume cover crop when dependent solely on atmospheric N_2_. The forage oat was used as the non-fixing reference plant. The β values used in this study were −0.79 ‰ and −0.36 ‰ for common vetch and fababean, respectively based recommendation of Unkovich et al. [11].

The fixed shoot N by the legume was determined using the Eq. (3)

(3) $$Fixed\;shoot\;N\;\left(kg\;N\;ha^{-1}\right)=\;Legume\;shoot\;N\;retention\;\left(kg\;N\;ha^{-1}\right)\times \;\frac{\%\;Ndfa\;}{100}$$

### 2.3 Soil measurements

Before the establishment of the experiments, eleven soil core samples were collected at each site and sectioned into five depth increments (0-10 cm, 10-30 cm, 30-60 cm, 60-90 cm, and 90-120 cm) to determine baseline soil physical and chemical properties. For each site, samples for each soil depth were composited to form a single sample per depth strata per site and were processed through a 2 mm sieve after oven-dried at 40°C until constant weight. The samples were analyzed and analyzed for bulk density, organic carbon (Walkey-black), pH (in CaCl_2_), and electrical conductivity using electrode, Colwell P and K, available N (NH_4_^+^–N + NO_3_–N), and particle distribution based on methods described in Rayment et al. (2011) at CSBP Soil and Plant Laboratory Ltd, WA, Australia. Undisturbed sub-samples were taken to determine the soil water characteristics of crop drained upper limit (DUL), and crop lower limit (LL15). The saturated sub-samples were used to characterize DUL (at 0.1 bar) and LL15 (at 15 bar) using Ceramic Pressure Plate Extractor (Agro-Ecosystems Soil Management Solutions, http://thinksoils.org/). In addition to the baseline soil samples, samples were collected at four different phases of the typified annual rotation: beginning fallow (cash crop harvest), cover crop sowing, cover crop termination, and end of fallow period (cash crop sowing) in each year. This allows estimating plant available water (mm) as the difference between the DUL and LL15 adjusted for later thickness and bulk density at each phase of the rotation.

Cover crop water use (CEP) was estimated as a residual of the soil water balance as expressed in Eq. (4):

(4) $$CEP\;\left(mm\right)=\;PAW_{s}-\;PAW_{t}+I+P$$

Where *PAWs* and *PAWt* are plant-available water at sowing and termination (mm), *I* is the supplementary irrigation applied (mm), and *P* is precipitation (mm). Subsequently, fallow efficiency (%FE) was determined as the proportion of precipitation received during the fallow period that is converted to plant-available water using Eq. (5).

(5) $$FE\;\left(\%\right)=\frac{Soil\;water\;at\;end\;of\;fallow\;\left(mm\right)\;-\;soil\;water\;at\;begining\;of\;fallow\;\left(mm\right)}{Water\;inputs\;during\;fallow\;\left(mm\right)}\;\times \;100$$

The soil mineral N accumulation (MIN) from cover crop termination to cash crop sowing (end fallow) was calculated as the relative change in soil mineral N as shown in Eq. 6.

(6) $$\text{MIN}\left(\text{kg}Nha^{--1}\right)=\text{SM}N_{\text{termination}}-\text{SM}N_{\text{cash}\text{crop}\text{sowing}}$$

## Ethics Statements

NA.

## CRediT Author Statement

**Ismail I. Garba:** Conceptualization, Methodology, Investigation, Data Curation, Formal analysis, visualization, writing – original draft preparation; **Alwyn Williams:** Conceptualization, Methodology, Writing – review & editing, Supervision, Funding acquisition.

## Declaration of Competing Interest

The authors declare that they have no known competing financial interests or personal relationships that could have appeared to influence the work reported in this paper.

## Data Availability

Datasets supporting the adoption of multifunctional cover crops related to soil water and nitrogen in water-limited environments (Original data) (UQ eSpace).

## Appendix Supplementary materials

Image, application 1
